# Risk Factors of Rheumatic Heart Disease in Bangladesh: A Case-Control Study

**DOI:** 10.3329/jhpn.v31i1.14751

**Published:** 2013-03

**Authors:** Baizid Khoorshid Riaz, Shahjada Selim, Md. Nazmul Karim, Kamrun Nahar Chowdhury, Shahabul Huda Chowdhury, Md. Ridwanur Rahman

**Affiliations:** ^1^Prime Minister's Office, Dhaka, Bangladesh;; ^2^Department of Endocrinology and Metabolism, BIRDEM, Dhaka, Bangladesh;; ^3^World Health Organization, Dhaka, Bangladesh;; ^4^National Centre for Control of Rheumatic Fever and Heart Disease, Dhaka, Bangladesh;; ^5^Department of Medicine, Bangabandhu Sheikh Mujib Medical University, Dhaka, Bangladesh;; ^6^Department of Medicine, Shaheed Suhrawardy Medical College, Dhaka, Bangladesh

**Keywords:** Case-control study, Rheumatic fever, Rheumatic heart disease, Risk factors, Bangladesh

## Abstract

Not all cases of rheumatic fever (RF) end up as rheumatic heart disease (RHD). The fact raises the possibility of existence of a subgroup with characteristics that prevent RF patients from developing the RHD. The present study aimed at exploring the risk factors among patients with RHD. The study assessed the risk of RHD among people both with and without RF. In total, 103 consecutive RHD patients were recruited as cases who reported to the National Centre for Control of Rheumatic Fever and Heart Disease, Dhaka, Bangladesh. Of 309 controls, 103 were RF patients selected from the same centre, and the remaining 206 controls were selected from Shaheed Suhrawardy Medical College Hospital, who got admitted for other non-cardiac ailments. RHD was confirmed by auscultation and colour Doppler echocardiography. RF was diagnosed based on the modified Jones criteria. An unadjusted odds ratio was generated for each variable, with 95% confidence interval (CI), and only significant factors were considered candidate for multivariate analysis. Three separate binary logistic regression models were generated to assess the risk factors of RF, risk factors of RHD compared to non-rheumatic control patients, and risk factors of RHD compared to control with RF. RF and RHD shared almost a similar set of risk factors in the population. In general, age over 19 years was found to be protective of RF; however, age of the majority (62.1%) of the RHD cases was over 19 years. Women [odds ratio (OR)=2.2, 95% CI 1.1-4.3], urban resident (OR=3.1, 95% CI 1.2–8.4), dwellers in brick-built house (OR=3.6, 95% CI 1.6-8.1), having >2 siblings (OR=3.1, 95% CI 1.5- 6.3), offspring of working mothers (OR=7.6, 95% CI 2.0-24.2), illiterate mother (OR=2.6, 95% CI 1.2-5.8), and those who did not brush after taking meals (OR=2.5, 95% CI 1.0-6.3) were more likely to develop RF. However, more than 5 members in a family showed a reduced risk of RF. RHD shared almost a similar set of factors in general. More than three people sharing a room also showed an increased risk of RHD (OR=1.9, 95% CI 1.0-3.4), in addition to the risk factors of RF. Multivariate model also assessed the factors that may perpetuate RHD among RF patients. Overcrowding (OR=2.4, 95% CI 1.2-4.7) and illiteracy (OR=2.4, 95% CI 1.1-5.2) posed the risk of RHD in the RF patients. The study did not find new factors that might pose an increased risk, rather looked for the documented risk factors and how these operate in the population of Bangladesh.

## INTRODUCTION

Globally, the prevalence of rheumatic fever (RF) and rheumatic heart disease (RHD) has declined sharply but, in developing countries, RF is still a leading cause of heart disease and, consequently, death in children and young adults (1,2). In 2005, it was estimated that over 2.4 million children aged 5-14 years were having RHD globally, and 79% of all these cases were from less-developed countries, such as Bangladesh (3). The prevalence of RF defined by the revised Jones criteria among children aged 5-15 years in rural Bangladesh was 1.2 (4). These are conservative estimates, especially if echocardiographic screening is used; the actual figures are likely to be substantially higher (5). RHD poses a huge burden on the health system in the resource-limited countries; the problems of RF and RHD will have to compete for limited resources with other more immediate and urgent health concerns, such as malnutrition, diarrhoeal diseases, and tuberculosis. If initiatives driven from evidence-based practice could be taken in the next few years, a substantial decrease in the prevalence of RF and RHD will occur in low-income countries too.

RHD is a well-documented sequel of RF, and not all RF patients develop RHD. There might be some risk factors to predispose patients in developing RHD. Identification of modifiable risk has the potential of easing of the instigation of prevention initiative. Efforts have been made by researchers to unearth the possible factors. A study by Meira and colleague followed up RF cases prospectively to see the sequel and reported several other risk factors which might also play a role. They identified education of mothers and recurrent RF episodes as factors contributing to RHD. An epidemiological survey of RF and RHD in South Africa provided some showcase evidence about several risk factors quite vividly. Among the white minority, who have experienced more privileged socioeconomic and healthcare status under the apartheid system, the prevalence of RF and RHD was low (6). Zaman *et al.* opined that protein-energy malnutrition is likely to be associated with RF (7). A study in Yugoslavia showed that socioeconomic issues, like flat dampness, living more than 2 persons per room, sleeping in bed with other persons, low education of mother, and undernourishment as risk factors of rheumatic fever were of lesser importance for persons with frequent sore throat compared to persons without frequent sore throat. They showed that there is a positive connection between host's propensity to clinical manifestation of throat infection and manifestation of rheumatic fever. The lesser the susceptibility, the more additional factors are needed for rheumatic fever to occur. The relative importance of socioeconomic factors in the occurrence of rheumatic fever depends on host's susceptibility to infection (8). Hence, it is worth exploring the factors that operate in the development of the disease. The aim of the present study was two-fold: to identify the risk factors of RF and to explore the risk factors of RHD among RF patients.

## MATERIALS AND METHODS

### Study setting and subjects

To detect four-fold or higher odds ratio, with the prevalence of RF (0.013%) among general population at 80% power and at a case-control ratio of one to three, 103 cases and 309 controls were required. Cases were consecutive RHD patients reporting to the outpatient department of the National Centre for Control of Rheumatic Fever and Heart Disease, the only national centre for RF and RHD in Bangladesh, where patients reach through referrals. Of the controls, 103 were RF patients selected from the same centre. The remaining 206 controls were selected from Shaheed Suhrawardy Medical College Hospital, who were admitted for other diseases, particularly non-cardiac ailments, including RF. RHD was diagnosed by careful cardiac auscultation and colour Doppler echocardiography. Patients in whom an organic murmur was detected clinically and confirmed successively with echocardiography were classified as having clinically-detected RHD. RF was diagnosed based on the modified Jones criteria. Primary data were collected by face-to-face interview of the patients by trained medical graduate research assistants during the period of hospital stay. Information regarding risk factors and risk behaviour was inquired with an effort to minimize the recall bias.

Data on the present state, diagnosis, and hospital-records were collected using a structured data-extraction form.

### Analysis of data

Analysis of data primarily focused on assessing the risk factors of RHD in patients with and without both RF and risk of RF among general population. Hence, the proportion of each of the factors was compared in three groups; association was sought for sociodemographic variables, parent factors, living condition, and oral health. Unadjusted odds ratio was generated for all the variables with 95% confidence interval, and only significant factors were considered candidate for multivariate analysis. Three separate binary logistic regression models were generated to assess risk factors of RF, risk factors of RHD compared to non-rheumatic control patients, and risk factors of RHD compared to RF control, adjusting for possible confounders. The statistical analysis was performed using the Stata® 10/IC software.

### Ethical approval

The protocol received ethical clearance from Bangladesh Medical Research Council.

## RESULTS

In total, 412 subjects were interviewed for the case-control study. The average age of the participants was 24.1±9 years, and 55% were women. Of the participants, about 48% were from urban area, about 16% were from semi-urban area, 6.8% were from slum area, and 30% were from rural area.

### Sociodemographic factors

Two-thirds of the RF patients were aged less than 20 years, around 38% of the RHD patients were aged less than 20 years, and among controls, the percentage was 16%. The proportions of women were more in both RF (OR=2.5) and RHD (OR=1.9) patients. The proportion of subjects with ≥5 members in the family was significantly more in both RF and RHD patients than the reference population. The proportion of subjects with ≥2 siblings and overcrowding (>3 persons sharing a living room) (OR=1.7) have been reported more among the RHD patients. The monthly family income and education level of patients showed no significant difference across the three groups (Table 1). The characteristics of parents, particularly education and occupation of both parents, were compared across the three groups. The numbers of mothers with low or no education and working mothers were more in RF and RHD patients compared to the reference group (p<0.05). Neither education nor occupation of fathers showed a significant difference across groups (p<0.05) (Table 2)

**T1able 1.** Sociodemographic variables in study subjects (n=413)

Variable	NRF	RF	RHD
	(n=207)	(n=103)	(n=103)
Age (years)			
<19	34 (16.4)	67 (65.0)	39 (37.9)
>20	173 (83.1)	36 (35.0)	64 (62.1)
χ^2^ (p value)	Reference	74.0 (p 0.001)*	17.5 (p 0.001)*
Sex			
Male	113 (54.6)	33 (32.0)	40 (38.8)
Female	94 (45.4)	70 (68.0)	63 (61.2)
OR (95% CI) for female	Reference	2.5 (1.5-4.2)*	1.9 (1.2-3.1)*
Family-size			
<5	45 (21.7)	40 (38.8)	35 (34.0)
≥5	152 (78.3)	63 (61.2)	68 (66.0)
OR (95% CI) for family-size ≥5	Reference	0.4 (0.26-0.7)*	0.54 (0.3-0.9)*
Number of siblings			
≤2	103 (49.8)	48 (46.6)	33 (32.0)
>2	104 (50.2)	55 (53.4)	70 (68.0)
OR (95% CI) for >2	Reference	1.1 (0.71-1.8)	2.1 (1.3-3.4)*
Persons per room			
≤3	99 (47.8)	57 (55.3)	36 (35.0)
>3	108 (52.2)	46 (44.7)	67 (65.0)
OR (95% CI) for >3 persons	Reference	0.74 (0.5-1.2)	1.7 (1.1-2.7)*
Family income (Tk) per month			
≤10,000	145 (70.0)	62 (60.2)	66 (64.1)
>10,000	62 (30.0)	41 (39.8)	37 (35.9)
OR (95% CI) >10,000	Reference	0.64 (0.4-1.1)	0.76 (0.46-1.2)

*Statistically significant; CI=Confidence interval; NRF=Non-rheumatic fever (control); OR=Odds ratio; RF=Rheumatic fever; RHD=Rheumatic heart disease; Figures in parentheses are percentages unless other-wise specified in the column-head

### Living condition and oral health

The study investigated the potential risk of living condition and oral health. According to our data, the majority (87.4%) of the RF and 78.6% RHD patients were the residents of urban or semi-urban area. The significant majority (64.1%) of the RF and 59.2% of RHD patients were living in *pucca* or semi-*pucca* houses, and the majority (75.7%) with RF and 65% with RHD were not using tubewell for drinking-water. Sleeping in floor was reported significantly more (14.6%) by the RHD (OR=2.5) patients than the reference group and RF patients. The use of toothpaste was not different between the RF and RHD patients from the reference group. However, brushing twice or more a day and habit of brushing after meal were found to be less among the RF patients than the reference group (Table 3).

### Rheumatic fever risk model

The risk factors of RF were assessed using the binary logistic regression model. The model explained 53.6% of the variability in the RF status. Females were more likely to develop RF (OR=2.2, 95% CI 1.1-4.3). The risk of RF was also high among urban residents (OR=3.1, 95% CI 1.2-8.4), and among people living in brick-built house (OR=3.6, 95% CI 1.6-8.1). Family-size showed an inverse relationship with the risk of RF; More than 5 members in a family showed a reduced risk of RF. However, the number of siblings (>2) appeared as a significant predictor. Offspring of working mothers (OR=7.6, 95% CI 2.0-24.2), illiterate mothers (OR=2.6, 95% CI 1.2-5.8), and those who did not brush after meal (OR=2.5, 95% CI 1.0-6.3) were more likely to develop RF (Table 4).

### Rheumatic heart disease risk model

Age, sex, place of residence, wall material at house, family-size, number of siblings, number of people sharing a living room, and education and occupation of mothers appeared as the significant predictors of RHD compared to the reference non-rheumatic control. A significant increased risk of RHD was evident in women (OR=2.2, 95% CI 1.2-4.2), urban residents (OR=2.0, 95% CI 1.2-7.0), living in brick-built house (OR=2.8, 95% CI 1.3-5.3), having siblings >2 (OR=4.4, 95% CI 2.2-8.7), child of working mother (OR=6.2, 95% CI 2.1-18.4), child of illiterate mother (OR=2.5, 95% CI 1.2-4.9), and overcrowding (OR=1.9, 95% CI 1.0-3.4). Age over 19 years (OR=0.1, 95% CI 0.1-0.3) and a large family-size (OR=0.5, 95% CI 0.2-0.9) appeared as the protective factors for RHD (Table 4).

**T1able 2.** Demographic characteristics of parents (n=413)

Parents' characteristics	NRF	RF	RHD
	(n=207)	(n=103)	(n=103)
Education of mother			
Secondary and above	78 (37.7)	25 (24.3)	16 (15.5)
Primary or less	59 (28.5)	30 (29.1)	35 (34.0)
Illiterate	70 (33.8)	48 (46.6)	52 (50.5)
χ^2^ (p value)	Reference	6.7 (0.033)*	11.7 (0.003)*
Occupation of mother			
Housewife	197 (95.2)	91 (88.3)	87 (84.5)
Working mother	10 (4.8)	12 (11.7)	16 (15.5)
OR (95% CI) for working mother	Reference	2.6 (1.1-6.2)*	3.6 (1.6-8.3)*
Education of father			
Secondary and above	113 (54.6)	60 (58.3)	46 (44.7)
Primary or less	94 (45.4)	43 (41.7)	57 (55.3)
OR (95% CI) for primary or less	Reference	1.2 (0.7-1.9)	0.7 (0.4-1.1)
Occupation of father			
Labour-intensive job	108 (52.2)	29 (28.2)	46 (44.7)
Moderate-activity job	43 (20.8)	31 (30.1)	27 (26.2)
Sedentary-activity job	56 (27.1)	43 (41.7)	30 (29.1)
χ^2^ (p value)	Reference	1.6 (p 0.06)	1.8 (p 0.41)

*Statistically significant; CI=Confidence interval; NRF=Non-rheumatic fever (control); OR=Odds ratio; RF=Rheumatic fever; RHD=Rheumatic heart disease; Figures in parentheses are percentages unless other-wise specified in the column-head

### Risk of rheumatic heart disease among rheumatic fever patients

Only age >19 years and overcrowding appeared as the significant predictors of RHD among the RF patients. Odds of being aged >19 years was 3.4 among the RHD patients compared to the RF patients. More than three persons sharing a room had a significant association with RHD (OR=2.4, 95% CI 1.2-4.7), and low attainment of education by mothers appeared as significant factor of RHD (OR=.2.4, 95% CI 1.1-5.2) (Table 4).

## DISCUSSION

In the present study, overcrowding and low level of education of mothers increased the risk of RHD among the RF patients. Urban residence, living in brick-built house, having three or more siblings, mothers working out of home, and overcrowding appeared as the significant risk factors of RHD in general in the case-control study. However, age over 19 years and a large family-size appeared as the protective factors for RHD.

### Rheumatic fever risk

In the current study, some socioeconomic, behavioural and environmental factors were found to play a pivotal role in altering the risk of people for developing RF and subsequently RHD. Around one-third of RF patients may not present any history of throat infection and may have negative cultures; there is usually an antibody response. Epidemiological studies, particularly in military institutions, have confirmed the associations between streptococcal infection and subsequent RF (9). However; there are several other factors that may alter the people's risk for developing the disease. Those factors may actually operate through increasing the risk of throat infection, or through any other mechanisms that are beyond the scope of the present study. Further detailed investigation is required to explore this. Our focus was to identify the factors that affect the individual's susceptibility. Our data confirmed that the age of over 19 years somehow is protective of having RF, and women are at a greater risk.

Among the RF patients, odds of being urban resident was 3.1 and of being inhabitant of brick-walled house was even higher (3.6). The urban people are likely to live in *pucca* houses. However, there must be a common factor or clue that increases the risk of RF. It may also point toward overcrowding. Contrary to the finding, sharing a room by more than three persons was not a significant predictor of RF, rather it was quite the reverse. A larger family-size appeared as a protective factor. Implication of such finding is that it is not the number of children or members in the family, rather it is the availability of persons to care for a child. The fact has been reflected with the observed association of the number of siblings with the risk of RF. Our data showed three-fold odds of having more than two siblings in RF patients. The career concept receives further concurrence as offspring of working mothers appeared as a highly-significant predictor of RF, with an odds ratio of 7.6. Another big factor that has been reported in most other studies is the education of mother (10). Most RF patients are the offspring of mothers with little or no education (OR=2.6). Education is expected to enable mothers to provide quality care efficiently. There might be explanations of such a finding which we did not consider in the present study. We inquired about living condition and have done meticulous assessment of the lifestyle factors to see any possible association with these factors with RF or RHD risk. A fundamental limitation of a case-control study is that it only generates measure of association for the factor anticipated by researchers.

**T1able 3.** Living conditions and oral health (n=413)

Factor	NRF	RF	RHD
	(n=207)	(n=103)	(n=103)
Residence			
Urban/semi-urban	118 (57.0)	90 (87.4)	81 (78.6)
Rural	89 (43.0)	13 (12.6)	22 (21.4)
OR (95% CI) for rural	Reference	0.2 (0.1-0.4)*	0.4 (0.2-0.6)*
Wall material			
Semi-*pucca* or *kaccha*	116 (56.0)	37 (35.9)	42 (40.8)
*Pucca*/brick	91 (44.0)	66 (64.1)	61 (59.2)
OR (95% CI) for *pucca* house	Reference	2.3 (1.4-3.7)*	1.8 (1.2-3.0)*
Water supply			
Supply or surface water	105 (50.7)	78 (75.7)	67 (65.0)
Tubewell/groundwater	102 (49.3)	25 (24.3)	36 (35.0)
OR (95% CI) for tubewell	Reference	0.3 (0.29-0.6)*	0.55 (0.34-0.9)*
Bed			
*Khat*	194 (93.7)	92 (89.3)	88 (85.4)
Floor	13 (6.3)	11 (10.7)	15 (14.6)
OR (95% CI) for floor	Reference	1.8 (0.72-4.1)	2.5 (1.16-5.8)
Dentifrice			
Conventional	14 (6.8)	14 (13.6)	12 (11.7)
Toothpaste	193 (93.3)	89 (86.4)	91 (88.3)
OR (95% CI) for toothpaste	Reference	0.46 (0.2-1.01)	0.55 (0.25-1.2)
Brush frequency			
Once daily or less	103 (49.8)	37 (35.9)	52 (50.5)
Twice daily or more	104 (50.2)	66 (64.1)	51 (49.5)
OR (95% CI) for twice or more	Reference	0.6 (0.4-0.9)*	1.0 (0.6-1.7)
Brush after meal			
No	189 (91.3)	75 (72.8)	87 (84.5)
Yes	18 (8.7)	28 (27.2)	16 (15.5)
OR (95% CI) for yes	Reference	0.2 (0.13-0.5)*	0.5 (0.3-1.1)

*Statistically significant; CI=Confidence interval; NRF=Non-rheumatic fever (control); OR=Odds ratio; RF=Rheumatic fever; RHD=Rheumatic heart disease; Figures in parentheses are percentages unless otherwise specified in the column-head

Developing countries are experiencing RF as a public-health problem now what developed countries faced earlier in the past century. The documented risk factors faced by the industrialized countries were: poverty, overcrowding, and reduced access to medical care. Nearly a similar risk factor was unveiled in the present study (11). In many parts of urban area, rapid industrialization has brought a population shift from rural to urban areas, leading to almost congested slums. This explains why the urban people are more prone to RF. Socioeconomic status was thought to have an influence on the epidemiology of acute RF. Between 1862 and 1962 in Denmark, the incidence of acute RF fell with the concomitant rise in the standard of living (12). A Serbian study identified the low educational level of mothers and home dampness as risk factors (13). In the same study, unemployment of parents and overcrowding were not significantly associated with acute RF. However, our data support significant association of overcrowding with RF and RHD risk. Children of working mothers in the study were found to be at greater risk of both RF and RHD. In our society, the employment status of mothers is not necessarily considered for economic implication, it is rather considered in line with care for babies.

**T1able 4.** Multivariate analysis (logistic regression) for risk factors of rheumatic fever and rheumatic heart disease

Factors included in model	Risk factors of RF in NRF patients		Risk factors of RHD in non-rheumatic controls		Risk factors of RHD in RF patient	
	OR (95% CI)	p value	OR (95% CI)	p value	OR (95% CI)	p value
Age (>19 years)	0.1 (0.03-0.1)	0.000*	0.1 (0.1-0.3)	0.000*	3.4 (1.7-6.9)	0.001*
Sex (female)	2.2 (1.1-4.3)	0.022*	2.2 (1.2-4.2)	0.012*	0.6 (0.3-1.3)	0.204
Residence (urban)	3.1 (1.2-8.4)	0.022*	2.0 (1.2-7.0)	0.015*	0.6 (0.2-1.7)	0.358
Wall material (brick)	3.6 (1.6-8.1)	0.034*	2.8 (1.3-5.3)	0.006*	0.8 (0.4-1.8)	0.610
Family-size (>5)	0.3 (0.2-0.7)	0.003*	0.5 (0.2-0.9)	0.037*	1.1 (0.5-2.3)	0.865
Number of siblings (>2)	3.1 (1.5-6.3)	0.002*	4.4 (2.2-8.7)	0.000*	1.4 (0.7-3.0)	0.332
Family income >10,000 Tk/month	0.9 (0.4-1.8)	0.678	0.8 (0.4-1.7)	0.623	1.0 (0.5-2.1)	0.949
Mother's education (Illiterate)	2.6 (1.2-5.8)	0.018*	2.5 (1.2, 4.9)	0.017*	2.4 (1.1, 5.2)	0.007*
Occupation of mothers (working)	7.0 (2-24.2)	0.001*	6.2 (2.1,18.4)	0.001*	1.0 (0.3-2.8)	0.948
>3 persons sharing a living room	1.2 (0.6-2.4)	0.639	1.9 (1.0-3.4)	0.046*	2.4 (1.2-4.7)	0.015*
Water supply (tubewell)	1.1 (0.4-2.6)	0.872	1.6 (0.7-3.6)	0.245	1.3 (0.6-3.0)	0.512
Bed (floor)	0.9 (0.3-3.4)	0.981	1.5 (0.5-4.1)	0.443	1.0 (0.3-2.8)	0.954
Dentifrice (toothpaste)	0.6 (0.2-1.7)	0.335	0.7 (0.2-1.8)	0.435	1.5 (0.5-4.3)	0.485
Brushing (≤1 times/day)	0.9 (0.4-1.7)	0.681	1.3 (0.7-2.4)	0.376	0.6 (0.6-3.0)	0.427
Brushing after meal (no)	2.5 (1.0-6.3)	0.042*	1.5 (0.6-3.9)	0.366	0.6 (0.2-1.5)	0.283
Nagelkerke R^2^	0.536		0.382		0.335	

*Statistically significant; CI=Confidence interval; OR=Odds ratio; RF=Rheumatic fever; RHD=Rheumatic heart disease; Figures in parentheses are CI for ORs

Oral health and its maintenance practice have been investigated for its possible link to the RF risk. As surrogate for oral health, the use of dentifrice (toothpaste), brushing frequency, and practice of brushing after meal were investigated. Among the factors considered, not brushing after meal appeared as the significant predictor, although tooth-brushing practice and the number of brushings did not appear as significant risk factors of RF.

We did not consider the nutritional status and dietary intake. Results of research showed that poor nutrition in early childhood plays a primary role in susceptibility to RF (14).

There are few studies on the relationship between socioeconomic factors and rheumatic fever in the populations where the burden of both socioeconomic deprivation and RF is still very high. A study in Bangladesh has investigated association of socioeconomic status and nutritional status with the risk of rheumatic fever. Authors concluded that RF was significantly associated with low income, poor living conditions and poor nutritional status in terms of low height-fo-age (15). Another study by the same authors carried out detailed nutritional investigation on rheumatic fever patients and drew similar inference (16). Another study among Bangladeshi children used serum albumin level as biochemical evidence to measure for nutritional status and found that patients with RF had statistically-significant lower albumin stores when compared with normal subjects (17). We considered monthly family income as a proxy for socioeconomic status, which almost steadily surrogates the nutritional intake, and no altered risk was found in different income groups.

### Risk of rheumatic heart disease

Not all people with RF have equal potential to develop RHD. Several factors, indeed, operate either solely or in combination in determining the individual's susceptibility to the development of RHD. Hence, along with the treatment of RF, several other factors should also be kept in mind for the determination of individual's risk. People with RF are much more likely to have subsequent episodes, and the recurrences may cause further damage to the cardiac valves. Thus, RHD steadily deteriorates in people who have repeated attacks of RF (18). Since RHD is a well-documented sequel of RF, we did not investigate the causal link. Our study focused on finding the factors that particularly make RF patients susceptible to RHD. In our study, a separate comparison was made between RF and RHD patients to determine the factors that may precipitate RHD among subjects with RF. Both the diseases share almost a similar set of factors that reflect the unidirectional causal link.

The present study revealed that the risk factor pattern for RHD was almost similar to that for RF. Age, sex, place of residence, wall material at house, family-size, number of siblings, number of persons sharing a living room, and occupation of mothers appeared as the significant predictors of RHD compared to the reference non-rheumatic controls. In the population, sharing of similar set of risk factors by RF and RHD may actually translate into the role of RF as an intermediate link of RHD causal pathway. Among the RF patients, overcrowding and low education of mothers appeared as significant predictors of an increased risk of RHD among offspring. In RHD risk, association of education of mothers can be viewed as the effect of quality of care by educated mothers. Oral health and its maintenance practice have also been investigated for its possible link to the RHD risk. None of the factors used as surrogate for oral health, e.g. the use of dentifrice (toothpaste), brushing frequency, and practice of brushing after meal appeared as significant risk factors of RHD. Although brushing habit after meal exert protective effect against RF, it's not associated with RHD risk. Poor oral hygiene possibly perpetuate streptococcal infection leading to RF, non-association of oral hygiene practice with RHD risk indicates that the pathogenesis of RHD following RF is probably independent of streptococcal infection. In animal model research, cardiac myosin has been defined as a putative auto-antigen recognized by auto-antibodies of RF patients. Endocardial infiltrate and their migration into the valve substance have been elegantly demonstrated in rats and mice (19).

Low risk of RHD in rural areas is probably due to less overcrowding. Areas where the prevalence of RHD is high, overcrowding is also the predominant factor. A study in Congo reported a high prevalence of RHD in urban areas compared to semi-urban area (1) where, on average, eight persons share a house. An Indian study reported a higher prevalence of RHD, unlike ours, in rural than in urban area. In our study, sharing a living room by >3 persons was found to be detrimental for RHD. Such practices pose as much as two-fold risk of RHD compared to healthy controls.

### Limitations

One limitation in the current investigation was that we used hospital-based sampling. Although a hospital-based consecutive sample cannot provide accurate prevalence data, these data nevertheless appear as a surrogate to describe the magnitude of RHD in the referral communities. Besides, our investigation did not focus on the prevalence, rather went for identifying the risk factor pattern. The role of oral health in RF or RHD risk has been investigated using the proxy variables. The link between oral hygiene practices and RF and RHD has not been much described in the disease process. The association of the brushing pattern with RHD risk highlights the need for future detailed investigation on this issue.

### Conclusions

RF and RHD share almost a similar set of risk factors in the population. Only overcrowding and low attainment of education by mothers pose RHD risk in RF patients. The study did not find new factors that might pose an increased risk, rather looked for the documented risk factors and how these operate in the population of Bangladesh.

## ACKNOWLEDGEMENTS

The study was funded by the Ministry of Health and Family Welfare of the Government of Bangladesh. The authors are grateful to Prof. Dr. Razia Sultana Mahmud, Director, National Centre for Control of Rheumatic Fever and Heart Disease and Dr. A.K.M. Mujibor Rahman, Director, Shaheed Suhrwardy Medical College Hospital, for their support. The authors also thank all the study subjects. They also thank the anonymous referees of the Journal for their thoughtful comments that have improved the presentation of the manuscript.

## References

[1] (1980). Community control of rheumatic heart disease in developing countries: 1 a major public health problem. WHO Chron.

[2] Agarwal BL (1981). Rheumatic heart disease unabated in developing countries. Lancet.

[3] Carapetis JR, Steer AC, Mulholland EK, Weber M (2005). The global burden of group A streptococcal diseases. Lancet Infect Dis.

[4] Ahmed J, Zaman MM, Hassan MMM (2005). Prevalence of rheumatic fever and rheumatic heart disease in rural Bangladesh. Trop Doct.

[5] Sadiq M, Islam K, Abid R, Latif F, Rehman AU, Waheed A (2009). Prevalence of rheumatic heart disease in school children of urban Lahore. Heart.

[6] Edginton ME, Gear JS (1982). Rheumatic heart disease in Soweto―a programme for secondary prevention. S Afr Med J.

[7] Zaman MM, Yoshiike N, Chowdhury AH, Nakayama T, Yokoyama T, Faruque GM (1998). Nutritional factors associated with rheumatic fever. J Trop Pediatr.

[8] Vlajinac H, Adanja B, Jarebinski M. Socio-economic factors and rheumatic fever occurrence (1989). Differences between patients with and without frequent sore throat. J Hyg Epidemiol Microbiol Immunol.

[9] Beggs S, Peterson G, Tompson A (2008). Antibiotic use for the prevention and treatment of rheumatic fever and rheumatic heart disease in children; report for the 2nd Meeting of World Health Organization's subcommittee of the Expert Committee of the Selection and Use of Essential Medicines.

[10] Kennell JH, Soroker E, Thomas P, Wasman M (1969). What parents of rheumatic fever patients don't understand about the disease and its prophylactic management. Pediatrics.

[11] Zabriskie JB. Rheumatic fever: the interplay between host, genetics, and microbe. Lewis A (1985). Conner memorial lecture. Circulation.

[12] Kaplan EL (1985). Epidemiological approaches to understanding the pathogenesis of rheumatic fever. Int J Epidemiol.

[13] Adanja B, Vlajinac H, Jarebinski M (1988). Socioeconomic factors in the etiology of rheumatic fever. J Hyg Epidemiol Microbiol Immunol.

[14] Coburn AF (1961). Susceptibility to rheumatic disease. J Pediatr.

[15] Zaman MM, Yoshiike N, Chowdhury AH, Jalil MQ, Mahmud RS, Faruque GM (1997). Socio-economic deprivation associated with acute rheumatic fever. A hospital-based case-control study in Bangladesh. Paediatr Perinat Epidemiol.

[16] Zaman MM, Yoshiike N, Chowdhury AH, Nakayama T, Yokoyama T, Faruque GM (1998). Nutritional factors associated with rheumatic fever. J Trop Pediatr.

[17] Zaman MM, Yoshiike N, Rouf MA, Haque S, Chowdhury AH, Nakayama T (1998). Association of rheumatic fever with serum albumin concentration and body iron stores in Bangladeshi children: case-control study. BMJ.

[18] Zabriskie JB, Freimer EH (1966). An immunological relationship between the group. A streptococcus and mammalian muscle. J Exp Med.

[19] Longo-Mbenza B, Bayekula M, Ngiyulu R, Kintoki VE, Bikangi NF, Seghers KV (1998). Survey of rheumatic heart disease in school children of Kinshasa town. Int J Cardiol.

